# Development of a health care utilisation data-based index for rheumatoid arthritis severity: a preliminary study

**DOI:** 10.1186/ar2482

**Published:** 2008-08-21

**Authors:** Gladys Ting, Sebastian Schneeweiss, Richard Scranton, Jeffrey N Katz, Michael E Weinblatt, Melissa Young, Jerry Avorn, Daniel H Solomon

**Affiliations:** 1 Division of Pharmacoepidemiology, Department of Medicine, Brigham and Women's Hospital, Harvard Medical School, 1620 Tremont Street, Suite 3030, Boston, MA 02120, USA; 2 Masschusetts Veterans Epidemiology Research and Information Center, VA Cooperative Studies Program, VA Boston Healthcare System, 150 South Huntington Avenue, Jamaica Plain, MA 02130, USA; 3 Division of Rheumatology, Immunology and Allergy, Department of Medicine, Brigham and Women's Hospital, Harvard Medical School, 75 Francis Street, Boston, MA 02115, USA

## Abstract

**Introduction:**

Health care utilisation ('claims') databases contain information about millions of patients and are an important source of information for a variety of study types. However, they typically do not contain information about disease severity. The goal of the present study was to develop a health care claims index for rheumatoid arthritis (RA) severity using a previously developed medical records-based index for RA severity (RA medical records-based index of severity [RARBIS]).

**Methods:**

The study population consisted of 120 patients from the Veteran's Administration (VA) Health System. We previously demonstrated the construct validity of the RARBIS and established its convergent validity with the Disease Activity Score (DAS28). Potential claims-based indicators were entered into a linear regression model as independent variables and the RARBIS as the dependent variable. The claims-based index for RA severity (CIRAS) was created using the coefficients from models with the highest coefficient of determination (R^2^) values selected by automated modelling procedures. To compare our claims-based index with our medical records-based index, we examined the correlation between the CIRAS and the RARBIS using Spearman non-parametric tests.

**Results:**

The forward selection models yielded the highest model R^2 ^for both the RARBIS with medications (R^2 ^= 0.31) and the RARBIS without medications (R^2 ^= 0.26). Components of the CIRAS included tests for inflammatory markers, number of chemistry panels and platelet counts ordered, rheumatoid factor, the number of rehabilitation and rheumatology visits, and Felty's syndrome diagnosis. The CIRAS demonstrated moderate correlations with the RARBIS with medication and the RARBIS without medication sub-scales.

**Conclusion:**

We developed the CIRAS that showed moderate correlations with a previously validated records-based index of severity. The CIRAS may serve as a potentially important tool in adjusting for RA severity in pharmacoepidemiology studies of RA treatment and complications using health care utilisation data.

## Introduction

Rheumatoid arthritis (RA) is an autoimmune disease characterised by pain, morning stiffness, joint swelling, deformity and functional impairments. Patients with RA have an increased risk of mortality and several adverse outcomes such as infections and cancer compared with those who do not have RA [1-4]. Several studies, however, suggest that complications in RA patients may not be attributable to the disease itself, but to the use of disease-modifying anti-rheumatic drugs (DMARD). For instance, tumour necrosis factor (TNF) α blocking agents have an association with specific types of infections and may be related to an excess risk of lymphomas and neurological complications [5-9]. Conventional DMARDs may also increase the incidence of lymphoma [10,11].

In studies that seek to determine the relationship between drug therapy and adverse events, disease severity is an important confounder. That is, disease severity is known to increase the risk of many adverse events and is probably associated with a higher likelihood of receiving more immunomodulating DMARDs. Failure to adjust for such confounding by indication can create false associations between the exposure and study outcome [12].

Health care utilisation ('claims') data are routinely collected for insurance and are commonly used in health services research [13,14]. Because adverse outcomes of RA are relatively rare, health care utilisation databases are an ideal source of information for studies of the relationship between DMARDs and adverse events such as cancer and infections. Thus, the development of an RA disease severity measure from claims merits high priority. We believe that health care claims data contain information such as physician visits, surgeries and laboratory tests that correlate with RA disease severity. Thus, to develop a claims-based severity index, we first created an RA medical records-based index of severity (RARBIS) from ratings by a Delphi panel on potential markers of RA severity commonly found in medical charts [15]. We then assessed the performance of the RARBIS in a cohort of Veteran's Administration (VA) patients and showed that the RARBIS correlated moderately well with RA treatment intensity and thus exhibited construct validity [16]. Next, we established the convergent validity of the RARBIS against a widely-used and accepted RA clinical measure, the Disease Activity Score (DAS28) [17]. The goal of the present study was to develop a claims-based severity index (claims-based index for RA severity [CIRAS]) using the previously validated RARBIS, not the DAS28. If validated as a measure of RA disease severity, the CIRAS may serve as a potentially important tool in adjusting for RA severity in pharmacoepidemiology studies of RA treatment and complications using health care utilisation data.

## Materials and methods

### Study population and data source

The study population consisted of 120 patients from the New England region of the VA Health System who had at least two recorded visits with a diagnosis of RA (International Classification of Disease-9-CM 714.0), at least two outpatient visits from hospitals within the New England VA Health System from July 1999 to June 2001 and had sufficient evidence of RA from their medical record. The VA maintains a comprehensive electronic medical records database containing information on demographic characteristics, surgical history, prescriptions, laboratory results, discharge summaries, radiology reports and progress notes. A review of the VA electronic medical records of the study population was conducted to obtain information on individual components of the RARBIS. The current study was approved by the VA Health System Human Subjects Committee.

### RA records-based index of severity

A records-based index of severity was developed based on ratings from a Delphi panel of six New England board certified rheumatologists of potential indicators of RA severity [15]. The potential indicators were divided into the following categories: radiological and laboratory results; surgeries; extra-articular manifestations; clinical and functional status; and medications (see Table 1). Indicators that were ranked by the panel as having strong or very strong associations with RA severity and are typically found in medical charts were incorporated into the RARBIS. Sub-scales and individual components of the RARBIS were weighted according to how strongly they were regarded by the panel as being correlated with disease severity. Because we wanted to develop an administrative-based severity score that could be used to study drug-outcome relationships, we created the RARBIS with the option to exclude the medication sub-scale.

**Table 1:** Rheumatoid arthritis medical records-based index of severity

**Sub-scale**	**Points**
**1. Surgery sub-scale:**	
C1–C2 fusion	3 points
Any hand joint	1 point
Any foot joint	1 point
Major joints (hips, knees, shoulder, elbow, wrist, ankle)	1 point each (max of 2)
Maximum score for category:	**5 points**
	
**2. X-ray sub-scale:**	
C1–C2 subluxation	3 points
Any erosions	1 point
Maximum score for category:	**4 points**
	
**3. Extra-articular manifestations sub-scale:**	
Vasculitis	1 point
Pulmonary nodule	1 point
Maximum score for category:	**1 point**
	
**4. Clinical status sub-scale:**	
Arthritis flares	
1	1 point
2 to 4	2 points
5 +	3 points
Worst physician global rating: "doing poor"	2 points
Functional status	
Unable to do hobbies	1 point
Unable to work	2 points
Unable to care for self	3 points
Hours of morning stiffness	
<1	0 point
1 to 4	1 point
>4	2 points
Maximum score for category:	**3 points**
	
**5. Laboratory sub-scale:**	
	
Rheumatoid factor titre > upper limit normal	1 point
Erythrocyte sedimentation rate > age/2 or C-reactive protein > upper limit normal or platelets > 450 K	1 point
Maximum score for category:	**2 points**
	
**Summary score for primary index**	**Maximum 15 points**
	
**6. Optional medication sub-scale:**	
Any of the following medications: hydroxychloroquine, gold, sulfasalazine	1 point
Any of the following medications: methotrexate, leflunomide	2 points
Any of the following medications: cyclophosphamide, azathioprine, cyclosporin, anakinra, adalimumab, etanercept, infliximab	3 points
Maximum score for category:	**3 points**
	
**Summary score for extended index:**	**Maximum 18 points**

Data on clinical status indicators (number of flares, physician global rating, functional and ambulatory status, presence of swollen joints, receipt of intra-articular and intramuscular injections, and hours of morning stiffness) and medication use from the VA medical records visit notes were collected for the chart review study period, 30 June 2000 to 30 June 2001. Data on medication use were derived from pharmacy records. We obtained information on surgical history (C1–C2 fusion and joint surgery), laboratory values (rheumatoid factor, erythrocyte sedimentation rate [ESR], C-reactive protein [CRP] and platelet counts), extra-articular manifestations (subcutaneous nodules and vasculitis) and X-rays (C1–C2 subluxation, erosions) from all available data in the medical record.

### Potential health care utilisation data indicators of RA severity

We extracted the following information from the VA databases: rehabilitation visits (physical and occupational therapy), rheumatology visits, plain radiographs (hand, wrist, foot, ankle and cervical spine), extra-articular manifestations (pulmonary, soft tissue nodules, Felty's syndrome and Sjogren's syndrome), number of inflammatory marker (CRP and ESR) tests, number of platelet counts and chemistry panels ordered, rheumatoid factor testing, joint surgery (hand, wrist, knee, foot, ankle, elbow, cervical spine and shoulder) and DMARD use. The administrative study data period included both the one-year (1 July 1999 to 29 June 2000) and two-year (1 July 1 1998 to 29 June 2000) period before the one-year chart review study period.

Each physical therapy and occupational therapy visit was counted as a rehabilitation visit. Tests for CRP and ESR were aggregated into one category. Tests performed on the same day counted as separate tests. The number of hand, wrist, foot, ankle and cervical spine radiographs were also added together into one category. Three methods were used to count the number of prescriptions in a given year. First, we counted the total number of prescriptions (including repeat prescriptions) for the following 10 medications: auranofin, aurothioglucose, azathioprine, cyclosporine, etanercept (Enbrel, Amgen), hydroxychloroquine, infliximab (Remicade, Centocor), leflunomide, methotrexate and sulfasalazine (adalimumab, abatacept and rituximab were not yet available for RA). For the second method, prescriptions for each DMARD were counted once and added to obtain the total number of different DMARDs. For the third method, synthetic DMARDs and biological DMARDs were counted separately. Prescription for each type of DMARD was counted only once and then added together to obtain the total number of different synthetic DMARDs and biological DMARDs.

### Statistical analyses

For each patient, scores were calculated for the RARBIS with and without the medication sub-scale using data from the medical chart review. Using Spearman non-parametric tests, the correlations between the RARBIS and various forms of administrative data variables were then analysed. Data taken from one year before the chart review and from two years before the chart review were examined.

We then built linear regression models with the RARBIS as the dependent variable and the administrative data variables as the independent variables using SAS (Cary NC) automated procedures and the forward, backward and stepwise selection methods to select the best model. Administrative data variables were entered into the model in the form that gave the highest Spearman correlation with the RARBIS. The inclusion criterion for model selection was p < 0.2.

We added the regression parameters based on each patient's covariate values using PROC Score (SAS, Cary NC) to calculate claims-based severity scores (with and without the medication variables) for each patient in the study cohort. Finally, we examined the correlation between the CIRAS and the RARBIS using the non-parametric Spearman correlation coefficient.

## Results

Characteristics of the study population are summarised in Table 2. The study cohort was predominantly male with a mean age of 71 years. During the chart review study period, most had no functional limitations (78%) and did not require a device or wheelchair for ambulatory purposes (66%). About one-half of the population had swollen joints, morning stiffness that lasted less than one hour, but did not have an arthritis flare. The mean score for the RARBIS with medications was 4.4 (range 0 to 11) and without medications was 3.0 (range 0 to 8).

**Table 2:** Patient characteristics based on information from the medical records review

	N (%) or mean (SD)
Age, years	70.6 (11.1)
No of rheumatology visits	3.0 (2.1)
Male	109 (91)
ACR functional classification	
Class I (no limitation)	93 (78)
Class II (self-care, working, no hobbies)	8 (7)
Class III (self care, not working, no hobbies)	6 (5)
Class IV (limited self care, bed-bound)	4 (3)
Ambulatory status	
Independent	79 (66)
With device	25 (21)
Wheelchair	5 (4)
Morning stiffness, hours	
<1	70 (58)
1 to 4	25 (21)
>4	8 (7)
Flares	
0	65 (54)
1	22 (18)
1 to 4	11 (9)
5+	3 (3)
Hospitalised	7 (6)
Swollen joints	64 (53)
Rheumatoid nodules	41 (34)
Vasculitis	1 (1)
Physician global: poor	7 (6)
Patient global: poor	11 (9)
Employed out of home	10 (8)
Received intraarticular injections	11 (9)
Received intramuscular injections	1 (1)
Presence of C1–C2 subluxation	2 (2)
Joint space narrowing	74 (62)
Joint erosions	61 (51)
Pulmonary nodule	11 (9)
RARBIS score (with medication sub-scale)	4.4
RARBIS score (without medication sub-scale)	3.0

ACR, American College of Rheumatology; RARBIS, rheumatoid arthritis records-based index of severity.

Table 3 provides the unadjusted Spearman correlations for the claims-based RA severity variables and the RARBIS with and without the medication sub-scale using data from one year before the chart review study period. The variables for rheumatology visits, inflammatory markers and other laboratory markers yielded the highest correlation with the RARBIS. In our analysis using administrative data from one year before the chart review period, the highest correlation between the RARBIS and the medication variable were obtained using the medication variable created from the sum of all DMARD prescriptions in method one. For both the RARBIS with and without medication scale, having data from two years before the chart review period did not substantially increase the Spearman correlation coefficients and, in some cases, even decreased the value of the coefficients (data not shown).

**Table 3:** Unadjusted Spearman correlations with the rheumatoid arthritis records-based index of severity (RARBIS) with and without medication sub-scale

	**RARBIS with medication sub-scale**		**RARBIS without medication sub-scale**	
**Claims-based variables**	**Correlation coefficient**	**p value**	**Correlation coefficient**	**p value**
Rheumatology visits	0.32472	< 0.001	0.1859	0.04
Rehabilitation visits	0.11249	0.22	0.19199	0.04
X-ray	0.07798	0.40	0.01623	0.86
Rheumatoid lung involvement	-0.02654	0.77	-0.0428	0.64
Felty's syndrome	0.16301	0.08	0.18168	0.047
Hand surgery	0.07074	0.44	0.05358	0.56
Number of inflammatory marker tests ordered	0.38775	<.0001	0.28664	0.002
Rheumatoid factor test	0.22267	0.01	0.22	0.02
Number of platelet counts ordered	0.29883	<0.0001	0.21888	0.02
Number of chemistry panels ordered	0.26246	0.004	0.15374	0.0936
Medication count	---	---	0.21497	0.0184

Table 4 presents the adjusted correlations between the claims-based RA severity variables and the RARBIS with and without the medication sub-scale with data from one year before the chart review study period. The forward selection models yielded the highest model R^2 ^for both the RARBIS with the medication sub-scale (R^2 ^= 0.31) and the RARBIS without the medication sub-scale (R^2 ^= 0.26). Using two years of data resulted in lower model R^2^s (data not shown).

**Table 4:** Adjusted correlations between claims-based variables and rheumatoid arthritis records-based index of severity (RARBIS) with and without medication sub-scale

	**RARBIS with medication sub-scale**	**RARBIS without medication sub-scale**
**Claims-based variables**	**Partial R**^2^	
Age and gender	0.08	0.05
Rheumatologist visits	0.01	N/A
Rehabilitation visits	0.01	0.04
Felty's syndrome	0.01	0.03
Number of inflammatory Marker tests ordered	0.14	0.08
		
Rheumatoid factor test	0.02	0.04
		
Number of platelet counts ordered	0.03	0.01
Number of chemistry panels ordered	0.01	0.02
		
**Model R**^2^	0.31	0.26

**Figure 1:**
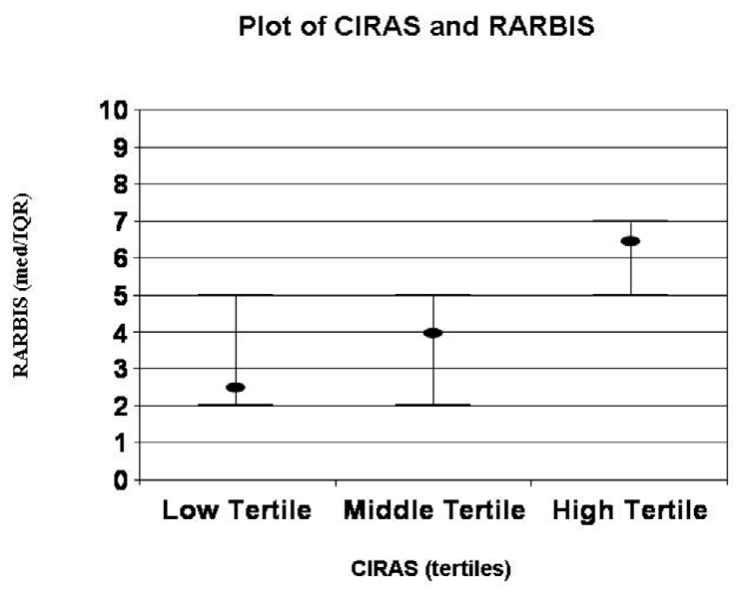
This plot illustrates the median and interquartile range for the tertiles of the claims-based index of rheumatoid arthritis severity (CIRAS). These values are plotted against the records-based index of rheumatoid arthritis severity (RARBIS). The CIRAS exhibits a moderate linear correlation with the RARBIS.

Table 5 includes the means and ranges for the CIRAS scores and the Spearman correlation coefficients between the CIRAS and the RARBIS. The CIRAS score with the highest correlation with the RARBIS included the following components: orders for inflammatory markers, rehabilitation visits, age and gender, rheumatoid factor, presence of Felty's syndrome, number of platelet counts and chemistry panels ordered, and rheumatology visits. Figure 1 is a graphic representation of this CIRAS score in tertiles versus the median and interquartile range for the RARBIS with medication sub-scale. Table 6 presents the suggested scoring method for the CIRAS.

**Table 5:** Claims-based index of rheumatoid arthritis severity (CIRAS) score (mean, range) and Spearman correlation of CIRAS score with rheumatoid arthritis records-based index of severity (RARBIS)

	**RARBIS with medication**	**RARBIS without medication**
**CIRAS (mean)**	4.38	3.03
**CIRAS (range)**	1.18–8.11	1.17–6.02
**CIRAS (Spearman, (pvalue))**	0.56 (<0.0001)	0.51 (<0.0001)

**Table 6:** Suggested scoring method for claims-based index of rheumatoid arthritis severity (CIRAS)

**Claims-based variables**	**Score**
**Age (continuous)**	**-0.066**
**Gender**	**-0.092**
0: male	
1: female	
**Number of inflammatory marker tests ordered**^a^	**0.60**
0: no	
1: yes	
**Rehabilitation visits**^a^	**0.69**
0: no	
1: yes	
**Rheumatoid factor test**^a^	**2.1**
0: no	
1: yes	
**Felty's syndrome**^a^	**2.3**
0: no	
1: yes	
**Number of platelet counts ordered**^a^	**0.42**
0: platelet count = 0	
1: platelet count = 1	
2: platelet count = 2	
3: platelet count = 3	
4: platelet count ≥ 4	
**Number of chemistry panels ordered**^a^	**-0.14**
0: chemistry panels = 0	
1: chemistry panels = 1	
2: chemistry panels = 2	
3: chemistry panels = 3	
4: chemistry panels = 4	
5: chemistry panels ≥ 5	
**Rheumatologist visits**^a^	**0.52**
1: number of rheumatology visits = 0	
2: number of rheumatology visits = 1, 2, 3, or 4	
3: number of rheumatology visits>4	
**Intercept**	**6.5**

^a^Time period for which data was captured for these variables is one year. Claims-based variables shown are included in the model selected from automated modeling procedures with rheumatoid arthritis records-based index of severity (RARBIS) as the dependent variable and potential claims-based variables as independent variables. Scores represent parameter estimates for these explanatory variables and can be used as weights when computing the CIRAS. To obtain an overall CIRAS score, multiply the value of each claims-based variable with its corresponding score and then sum all the scores and the value for the intercept.

## Discussion

We developed a claims-based RA severity index (CIRAS) that demonstrated moderate correlation with a previously validated medical records-based index, the RARBIS. The RARBIS has been previously shown to have good construct validity and moderate convergent validity with the DAS28 [16,17]. Because health care utilisation databases are a valuable source of data for studying health outcomes, other investigators have also used medical records-based indices to create indices for administrative databases. For instance, Deyo and colleagues adapted the Charlson Comorbidity Index, a well-validated index designed for medical records, so that *International Classification of Diseases, Ninth Revision *codes could be used to calculate the Charlson Comorbidity Index from administrative data [18]. Components of the administrative-based index we developed for RA include orders for inflammatory markers, number of platelet counts and chemistry panels ordered, rheumatoid factor, rehabilitation visits, age and gender, presence of Felty's syndrome and number of rheumatology visits. If the CIRAS is found to be valid in other populations, then it might be used to partially adjust for an important confounder, disease severity, in claims-based epidemiology studies. In our analysis, we used data taken from one and two years before the chart review study period. However, using two years of data resulted in lower R^2 ^and Spearman correlation values. Including another year of older data might have caused a dilution effect. Additionally, to compute scores on the CIRAS, we used weights from the regression models with the RARBIS. Other methods of weighting could have been chosen, for example, assigning a value of one to administrative variables that had significant correlations with the RARBIS. However, we believe that the method we selected, using beta coefficients as weights, better captures the relationship between the CIRAS and the RARBIS.

Because administrative data are collected primarily for reimbursement purposes, some question the use of claims data for clinical research regarding disease severity [19]. However, administrative data are gaining increasing acceptance in health care research, because they represent typical populations, contain large cohorts of patients with given conditions and are readily available. We also demonstrate in the present study that indicators of RA severity from claims data are moderately well related to clinical indicators of RA severity. Thus, it is possible to capture RA disease severity to some degree in claims data. Other proxies for severity of illness measures using claims data such as the diagnosis related group, the all patients refined diagnosis related group and the *International Classification of Diseases Ninth Revision*-Based Illness Severity Score have been developed [20]. Unlike the CIRAS, these other measures are not specific to RA.

The present study has important limitations. Our data source for this study was the New England VA Health system. The VA's population is mostly older men. Older male patients with RA may not represent typical RA patients. This highlights the need to consider these findings as preliminary and requiring replication in other settings. Additionally, data from the VA might be gathered differently from other health care systems, again highlighting the preliminary nature of our findings. However, because the VA contains rich data from both medical record and health care utilisation databases, it is a unique and ideal data source for our analysis. Additionally, the RARBIS, which we used to create the CIRAS, was developed using standard nominal group technique methods, followed by assessing its convergent validity with the DAS28. However, the DAS28 is a measure of disease activity not disease severity. While disease activity is an important component of disease severity, it is not the same. Currently, there is no standard RA disease severity measure.

In our cohort of 120 VA patients, the CIRAS showed moderate correlations with a validated medical records-based index and can be used for improved adjustment of RA disease severity in claims data studies. We do not believe that the value of the CIRAS will be limited to the VA population. We plan on assessing its validity in other populations, such as Medicare patients, and will examine its ability to adjust for confounding and predictive validity for outcomes known to be associated with severe RA, such as future joint surgeries, higher medical care costs and use of combination DMARDs. Additionally, we will explore whether different variations of the CIRAS should be used depending on the study outcome of interest. Ultimately, the CIRAS may be an important methodological tool for researchers studying RA treatment and complications using health care utilisation data, but further tests need to be conducted in other populations.

## Conclusion

We developed a claims-based severity index (CIRAS) from a previously validated medical records-based index (RARBIS). The CIRAS can potentially be used for improved adjustment of RA severity in studies of RA medication use and adverse outcomes using claims data, but future studies should examine its validity in other populations.

## Abbreviations

ACR = American College of Rheumatology; CIRAS = claims-based index of rheumatoid arthritis severity; CRP = C-reactive protein; DAS = disease activity score; DMARD = disease modifying anti-rheumatic drug; ESR = erythrocyte sedimentation rate; RA = rheumatoid arthritis; RARBIS = rheumatoid arthritis records-based index of severity; TNF = tumour necrosis factor; VA = Veterans Administration

## Competing interests

The authors declare that they have no competing interests.

## Authors' contributions

GT analysed the data and drafted the manuscript. SS provided support on the statistical analyses, interpretation of data and helped edit the manuscript. RS provided access to the data and helped edit the manuscript. JNK and MEW provided advice on the conceptual design and helped edit the manuscript. MY provided access to the data and helped edit the manuscript. JA contributed conceptual advice and helped edit the manuscript. DHS provided conceptual design, analytic support, access to the data and helped edit the manuscript.
